# Translation from murine to human lung imaging using x-ray dark field radiography: A simulation study

**DOI:** 10.1371/journal.pone.0206302

**Published:** 2018-10-29

**Authors:** Janne Vignero, Nicholas W. Marshall, Greetje Vande Velde, Kristina Bliznakova, Hilde Bosmans

**Affiliations:** 1 Department of Imaging and Pathology, KU Leuven, Leuven, Belgium; 2 Department of Radiology, UZ Leuven, Leuven, Belgium; 3 Laboratory of Computer Simulations in Medicine, Technical University of Varna, Varna, Bulgaria; Emory University School of Medicine, UNITED STATES

## Abstract

Recent studies on murine models have demonstrated the potential of dark field (DF) x-ray imaging for lung diseases. The alveolar microstructure causes small angle scattering, which is visualised in DF images. Whether DF imaging works for human lungs is not a priori guaranteed as human alveoli are larger and system settings for murine imaging will probably have to be adapted. This work examines the potential of translating DF imaging to human lungs. The DF contrast due to murine and human lung models was studied using numerical wave propagation simulations, where the lungs were modelled as a volume filled with spheres. Three sphere diameters were used: 39, 60 and 80 μm for the murine model and 200, 300 and 400 μm spheres for the human model. System settings applied for murine lung response modelling were taken from a prototype grating interferometry scanner used in murine lung experiments. The settings simulated for human lung imaging simulations combine the requirements for grating interferometry and conventional chest RX in terms of x-ray energy and pixel size. The DF signal in the simulated murine model was consistent with results from experimental DF data. The simulated linear diffusion coefficient for medium alveoli diameters was found to be (1.31±0.01)⋅10^−11^ mm-^1^, 120 times larger than those of human lung tissue ((1.09±0.01)⋅10^−13^ mm^-1^). However, as the human thorax is typically a factor 15 times larger than that of murine animals, the overall DF effect in human lungs remains substantial. At the largest lung thickness and for the DF setup simulated, human lungs have an estimated DF response of around 0.31 and murine lungs of 0.23. Dark field imaging can therefore be considered a promising modality for use in human lung imaging.

## Introduction

In recent years x-ray dark field (DF) imaging has evolved into a promising tool for lung imaging [1] based on its sensitivity to the air-tissue transitions in the alveoli. Increased detectability of emphysema [2–5], fibrosis [6,7] and pneumothoraxes [8,9] has been demonstrated. Dark field images can be obtained using various methods [10–12], but the present study focuses on grating interferometry based techniques (GI) [13] as most DF lung applications in the literature make use of this implementation. More specifically, Talbot interferometry was considered, in which two gratings (G_1_ and G_2_) are used to respectively create and measure a high frequency Talbot fringe pattern [14]. Nevertheless, the main outcome of this work will also be applicable for different dark field implementations. In GI the DF signal, *df*, is mainly related to the visibility loss of the Talbot fringe pattern due to incoherent interactions in the object, or

$$df=v_{obj}/v_{ref}$$ (1)

where the visibility *v* is related to the relative amplitude of the fringe pattern in a pixel. Every acquisition using a GI method yields, besides a DF image, an attenuation and a differential phase image, corresponding to respectively the mean intensity and the phase shift of the fringe pattern [14]. These images contain additional information on the imaged object, but are not discussed here.

The DF response to different materials is complex and is more difficult to predict than attenuation and differential phase imaging, where the contrast can be directly calculated from the material dependent parameters *β* and *δ* respectively [14]. In DF, the logarithm of *df* scales linearly with the thickness of the material. This has been expressed as [15]:

$$-\log\left(df\right)=\frac{2\pi^{2}d^{2}}{p_{2}^{2}}\cdot \epsilon \;t$$ (2)

where *t* is the thickness of the material and *ϵ* the material specific ‘linear diffusion coefficient’. In the same equation, *d* is the G1-to-G2 distance and *p*_2_ the period of the fringe pattern. The link between *ϵ* and the material compositions and structures is complex and has been studied extensively over the years [16–21]. The fundamental idea is that *ϵ* is low for homogenous materials and high for materials with a large number of density transitions. Both the quantity and magnitude of the density fluctuations, the *δ*,*β* material values and the length scale of the fluctuations play a role in the magnitude of the measured DF signal [16–18]. For a material composed of randomly distributed spheres of diameter *S*, covering a given volume fraction, Lynch et al. [18] calculated and demonstrated that the *ϵ* factor is a maximum when *S* = 1.8 ⋅ *d*_*auto*_, with

$$d_{auto}=\lambda \frac{d}{p_{2}},$$ (3)

the autocorrelation length. In *π*-phase shift Talbot setups, the following equation holds [22]:

$$d\approx m\frac{p_{2}^{2}}{2}/\lambda ,$$ (4)

Or

$$d_{auto}\approx \frac{mp_{2}}{2}$$ (5)

with *m* an odd integer. At the first Talbot order (m = 1) and for *p*_2_ = 2 *μ*m, *d*_*auto*_ approximates 1 *μ*m meaning that *ϵ* is maximized for 2 *μm* diameter spheres and decreases for larger spheres.

The majority of the pulmonary DF imaging feasibility studies performed today, use murine models [2,4–7,23–26]; the translation to human lung imaging is therefore not obvious as the DF response strongly depends on microscopic characteristics of the lung tissue. In humans, the alveoli typically range between 200 and 400 *μ*m, which is a factor of five larger than in mice (39–80 *μ*m) [27], and therefore, following Eq (5), a drop in the linear diffusion coefficient is expected. At the same time, the human thorax is thicker and the overall DF signal loss would be larger if *ϵ* would have the same value (see Eq (2)). For DF lung imaging to be applicable in humans, it should be sensitive to structural changes in the lung. This sensitivity is maximized when the DF signal at the largest lung thicknesses along the beam direction, is a minimum without being saturated. As far as we know, the translation from murine to human DF lung imaging has not been documented and comparative DF lung imaging has not been modelled. Given some initial studies on porcine lungs have been successful [9,28], a certain degree of sensitivity is expected for human lungs as well. Porcine lungs have alveolar sizes and lung volumes of the same order of magnitude as humans. Still, quantitative modelling is useful to investigate the extent to which this method can be applied and which system adaptations may be required and to understand the role of the different features attributing to the overall dark field signal measured. This leads to the question addressed in this work: ‘how well will dark field lung imaging perform for human lungs?’. This study estimates the impact of increased alveoli sizes on the DF contrast using numerical wave propagation simulations. Murine response is evaluated using the settings of a prototype GI system. Experimental data for this system are then used to validate simulations of the murine model. Second, clinical geometries similar to those in conventional chest x-ray are then applied in the evaluation of human lung DF response.

## Materials and methods

Numerical simulations were used to predict the average DF signal for murine and human lung models. The sizes of the alveoli and the dimensions of the lung were taken into account, as well as the acquisition settings of a typical chest PA imaging protocol. The linear diffusion coefficient for murine and human lung was then estimated based on the DF response of six thicknesses, *t*, using Eq (2).

### Simulation framework

The simulations were performed using a numerical wave propagation framework. In the framework the x-ray wave was modeled as a 2 dimensional grid (*x*,*y*) of complex wave functions and evaluated throughout different positions in the system. As the DF signal depends on microscale variations, fine sampling of the wave function is necessary. The index ′s′ denotes the finely sampled grid, without index refers to pixel sampling.

In the simulation platform, a plane wave interacts first with the object (i.e. the lung model) and the phase grating G_1_, followed by propagation in free space to the attenuation grating G_2_, where the (averaged) pixel intensity is recorded by the x-ray detector. By stepping G_2_, the intensity pattern is sampled and a stack of N_G2_ projections is created [14]. Via a fast Fourier transform, the average pattern as a function of the position of G_2_ (*x*_*G*2_) can be retrieved [14]. The wave function at the detector is given by

$$\psi (x_{s},y_{s},x_{G2})=\left[F^{-1}\{F\{\psi_{0}(x_{s},y_{s})\cdot O(x_{s},y_{s})\cdot G1(x_{s},y_{s})\}\cdot {\hat{H}}_{d}(u_{s},v_{s})\cdot F\{F_{gauss,G_{0}}(x_{s},y_{s})\}\}\right]\cdot G2(x_{s},y_{s},x_{G2})$$ (6)

In our implementation, a monochromatic plane wave was assumed, making *ψ*_0_(x_s_,y_s_) equal to unity. The transmission through the object is described by $O(x_{s},y_{s})={\exp\left(\frac{i2\pi }{\lambda }\int \delta (x_{s},y_{s},z)dz-\frac{2\pi }{\lambda }\int \beta (x_{s},y_{s},z)dz\right)}^{}$, with *λ* the wave length and *z* the propagation direction. The coefficients δ,β are related to the refractive index of the object via *n* = 1 − *δ* + *iβ* [29]. The grating G_1_ introduces a periodic phase shift of *π*, or G1(x_s_,y_s_) = exp(*iπΠ*(*x*_*s*_,*y*_*s*_)), where Π denotes a rectangular pulse function. Propagation over a distance *d* in free space is then simulated by a convolution with the free wave propagator *H* and in Fourier space $\hat{H}(u_{s},v_{s})={\exp\left(-i\pi d\lambda (u_{s}^{2}+v_{s}^{2})\right)}^{},$ where (u_s_,v_s_) are the spatial frequencies corresponding to the spatial dimensions [30,31]. Blurring due to the finite G_0_ apertures is included by filtering the wave function with a Gaussian kernel with standard deviation equal to the scaled G_0_ aperture width ($F_{gauss,G_{0}}(x_{s},y_{s})$). Lastly, G2(x_s_,y_s_,x_G2_) = Π(x_s_,y_s_,x_G2_), represents the absorption grating G_2_, which is evaluated at its fractional distances *x*_*G*2_.

The pixel values are generated by integrating the finely sampled intensity of *ψ* over the pixel area (*A*_*P*_) and scaled to realistic values via an x-ray dose dependent calibration factor (*S*_*PV*_). Here, S_PV_ is the average detector pixel value for a given air kerma (EAK), normalized to the integrated intensity of a reference *ψ* function without object in place. S_PV_ was calibrated for the prototype TLI system as a function of EAK, measured at 5.6 cm from the G_1_ grating (the centre location of the object). By assuming a linear relationship between detector pixel value and EAK, the detector pixel value at any EAK can be estimated. Blurring due to both the finite focal spot size and the detector is included by Fourier filtration in the spatial frequency domain. For the former, a Gaussian kernel (*F*_*gauss*_) with a standard deviation equal to the scaled focal spot size of the source was used while detector blurring was applied using the presampling modulation transfer function. Lastly, noise of the correct texture and magnitude was added to each projection, resulting in the final pixel intensities *I*(*x*,*y*,*x*_*G*2_).

$$I(x,y,x_{G2})={F^{-1}\{F\{\sum_{A_{P}}{\left|\psi (x_{s},y_{s},x_{G2})\right|}^{2}\cdot S_{PV}}^{}\}\cdot MTF(u,v)\cdot F\{F_{gauss,FS}(x,y)\}\}+N(x,y,x_{G2})\;\sigma (x,y,x_{G2})/\sigma_{N}$$ (7)

Where MTF(u,v) for the x-ray detector was determined from experimental data using an implementation of a slanted edge technique [32,33]. The noise, $N(x,y,x_{G2})=F^{-1}\left\{F\{R(x,y,x_{G2})\}\cdot \sqrt{NPS(u,v)}\right\}$, is modelled as a matrix of normally distributed random values (*R*) with zero mean and unit variance, filtered by the square root of the noise power function (NPS) [34]. Also the NPS was determined from experimental data using the approach described in [35]. Due to the Poisson nature of the noise, the expected noise magnitude in each pixel σ(x,y,x_G2_) is equal to the square root of the measured intensity in the corresponding pixel. To ensure a unit variance in *N*, the noise image is scaled with a factor 1/*σ*_*N*_, with *σ*_*N*_ the average standard deviation of *N*.

This calculation is done twice, once with object in place and once without object. Following [14] it is then possible to produce transmission, differential phase and dark field images. Here only the dark field images were considered, although accurate transmission and differential phase images are also generated. Due to the very fine sampling of *ψ*(x_s_,y_s_,x_G2_), these calculations are computationally expensive and creating large field of view images is time consuming. However, this did not restrict this study as large fields of view were not required.

The periods of the G_1_ and G_2_ grating were set to match those of a prototype Talbot-Lau GI system in our centre, with specified pitches of 3.901 *μ*m and 2.000 *μ*m. Other parameters depended on the study and are tabulated in Table 1. The G_1_-to-G_2_ distance was set at the first Talbot distance with characteristic values that depend on the x-ray energy (Eq 4). A high x-ray exposure (10^3^ mGy) was used to minimize the influence of noise, and therefore accurate modelling of system visibility was not required. If a future study requires the correct magnitude then this can be achieved by empirically adjusting the transmission through G_2_.

**Table 1:** Summary of settings for the murine and human modelling studies. The mean x-ray energy was set as the design energy of the system.

	Murine		Human	
Diameter alveoli S [*μ*m]	39–80		200–400	
Maximum AP thickness lung [cm]	1.1		20	
Pixel size [*μ*m]	100		150	
kVp (mean energy [keV])	40 (27.7)		120 (64.5)	
*d* [cm]	4.47		10.4	
*δ*, *β* air	2.71 × 10^−10^	1.42 × 10^−13^	9.02 × 10^−11^	4.27 × 10^−14^
*δ*, *β* muscle tissue	3.10 × 10^−07^	1.78 × 10^−10^	1.04 × 10^−07^	5.07 × 10^−11^

### Lung models

The lung was modelled as a compartment of spheres [36,37]. The centres of the spheres were generated randomly and the sphere volumes were allowed to overlap. Creating non-overlapping spheres is computationally expensive for large volume fractions and given that the objects are projected onto a single image plane, has little advantage. In the literature [37] alveoli are represented by hemispheres and overlapping spheres, may be even closer to reality. The linear diffusion coefficient, for both murine and human lungs, was estimated in the case of a minimum, maximum and intermediate diameter alveoli. The true lung response, characterized by a distribution of alveoli sizes, is expected to lie within the range of the estimated *ϵ* values. The volume fraction of the spheres was set to 0.56, a bit lower than the volume fraction in the case of random close packing of spheres (0.64) [38]. In order to fit Eq (2) accurately, the DF contrast was estimated for six AP thicknesses. For each thickness, the average DF signal in the whole field of view was calculated. The computation was repeated 10 times with new random generation of the sphere locations, giving a final average value and standard deviation for each thickness. Using Eq (2) the linear diffusion coefficient was then calculated (GraphPad Prism version 5.04 for Windows, GraphPad Software, California, USA). Values for the murine and human lung total thicknesses were estimated from CT data (Table 1).

The *δ* and *β* values for lung tissue were estimated using Henke et al [29] and NIST data [39,40], while compositions were taken from the ICRP110 [41]. The tissue in between the spheres was assumed to be made of muscle tissue and the spheres themselves were made of air (with zero wall thickness). The compressed lung tissue tabulated in ICRP110 is a mixture of both lung and air tissue and was thus not considered. The applied parameters and settings are summarized in Table 1.

#### Murine

The murine lung model was evaluated for alveoli diameters of 39, 60 and 80 *μm* [27]. The DF response was estimated for AP thicknesses of 1.8, 3.6, 5.4, 7.2, 9.0 and 10.8 mm. Every 1.8 mm of lung consisted of on average of respectively of 325, 89 and 38 spheres per detector pixel. The simulated field of view was 5 × 5 pixels of 100 *μ*m.

#### Human

The human lung model consisted of 200, 300 or 400 *μm* diameter spheres [27]. The AP thicknesses evaluated are thicker than in the murine model, i.e. 25, 50,75 100, 125 and 150 mm. This resulted in 75, 22 and 9 spheres per pixel per 25 mm thickness for the different alveoli sizes. In conventional chest-RX the voltage of the tube is typically set to 120 kVp, corresponding to an intensity-weighted mean energy of 64.5 keV for 3 mm Al system filtration, 1800 mm air and 90 mm PMMA [42]. The design energy of the setup was assumed to equal the intensity-weighted mean energy. Higher mean energy implies a longer distance *d* and is also linked with lower x-ray *δ* and *β* coefficients (see Table 1). For typical pixel sizes in chest x-ray (120–150 *μ*m), the alveoli exceed the pixel sizes and the field of view was increased to 10 by 10 pixels.

### Experimental validation

To validate the simulation model, the *ϵ* results from the murine simulations were compared to experimental data acquired using the prototype GI setup (Carestream Health, USA) installed at our small animal research centre (MoSAIC, KU Leuven). The most straightforward approach would use in-vivo GI CT datasets of a mouse, however this is not possible with this particular system. An CT acquisition takes up to four hours for sufficient signal-to-noise levels and it is not possible to keep the animals sedated over such long periods. To ensure normal lung inflation when measuring the DF response, a dark field projection scan was used in combination with a *μ*CT scan (SkyScan 1278, Bruker, Kontich, Belgium), both made in-vivo. This allows the lung thickness (from the *μ*CT) to be combined with the DF response, to estimate the *ϵ* experimentally. Three small ROIs (1 x 1 mm^2^) were selected in the dark field projection image of the mouse lung with low, medium and high DF response. Under the assumption that the main contributor to the DF signal contrast is due to small angle scattering in the lung, the dark field signal was then related to the thickness of the lung at the three corresponding positions in the *μ*CT scan. To reduce the influence of mouse fur on the DF data, ultrasound gel was applied to limit the hair-air transitions. The experiments were conducted under the institutional guidelines for animal welfare and approved by the KU Leuven ethical committee for animal research.

## Results

**Fig 1:**
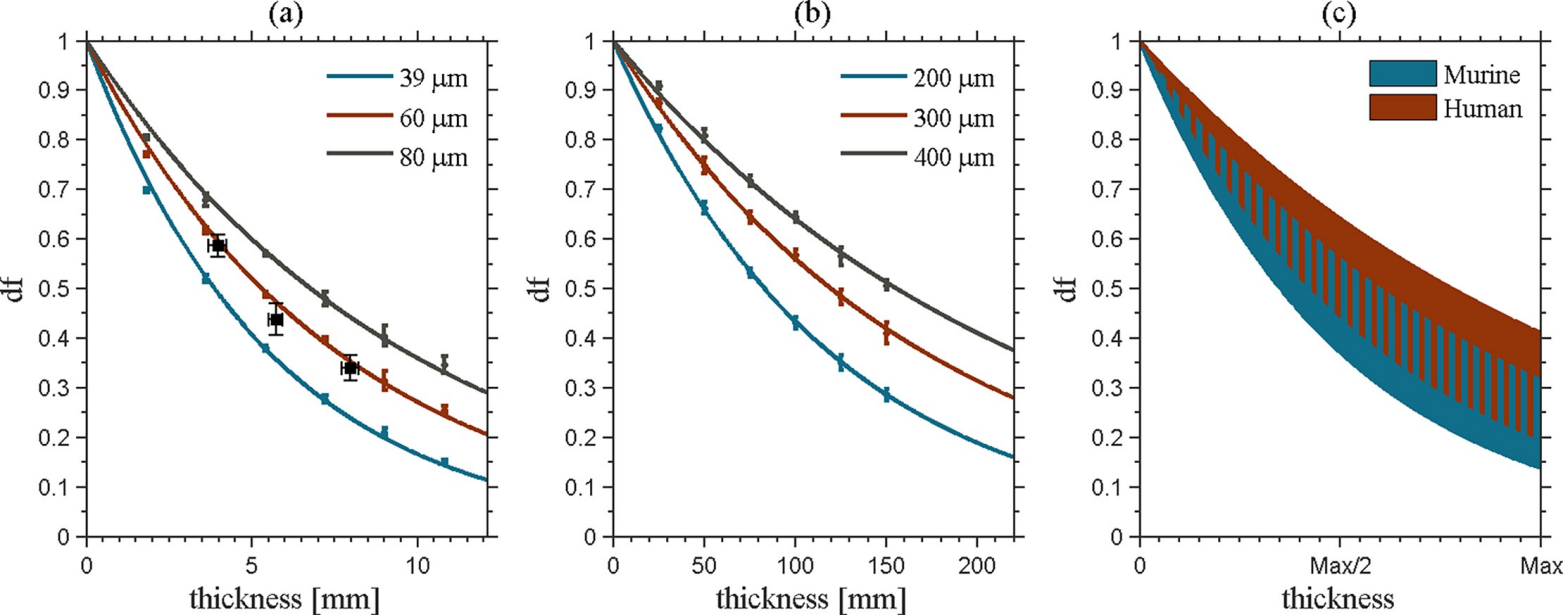
The dark field signal as a function of thickness for the murine (a) and the human model (b). In (c) a comparison is plotted as a function of relative thickness, ranging from 0 thickness to maximal thickness. The range of murine and human dark field response overlap. In (a), experimental data (black squares) validate the simulated results of the murine model.

Fig 1(A) shows DF response as a function of thickness for the mouse model for the three different alveoli sizes. Using the simulations, linear diffusion coefficients of (1.97±0.01)⋅10^−11^ mm^-1^, (1.31±0.01)⋅10^−11^ mm^-1^ and (1.02±0.01)⋅10^−11^ mm^-1^ were found for respectively 40, 60 and 80 *μ*m diameter murine alveoli. Meanwhile, for the human model (Fig 1(B)), *ϵ* equalled (1.57±0.01)⋅10^−13^ mm^-1^, (1.09±0.01)⋅10^−13^ mm^-1^ and (8.39±0.01)⋅10^−14^ mm^-1^ for respectively 200, 300 and 400 *μ*m diameter alveoli. For the medium sized alveoli, the linear diffusion coefficient is a factor 120 smaller in human lung compared to mouse lung tissue. However, as the lungs of humans are much thicker, at the largest thickness, a similar DF contrast is created in the human model compared to the murine model (see Fig 1(C)). Comparing DF signals at the respective maximum thicknesses, the murine DF signal is around 0.23, while the human signal is around 0.31. The human lungs therefore produce a comparable contrast.

**Fig 2:**
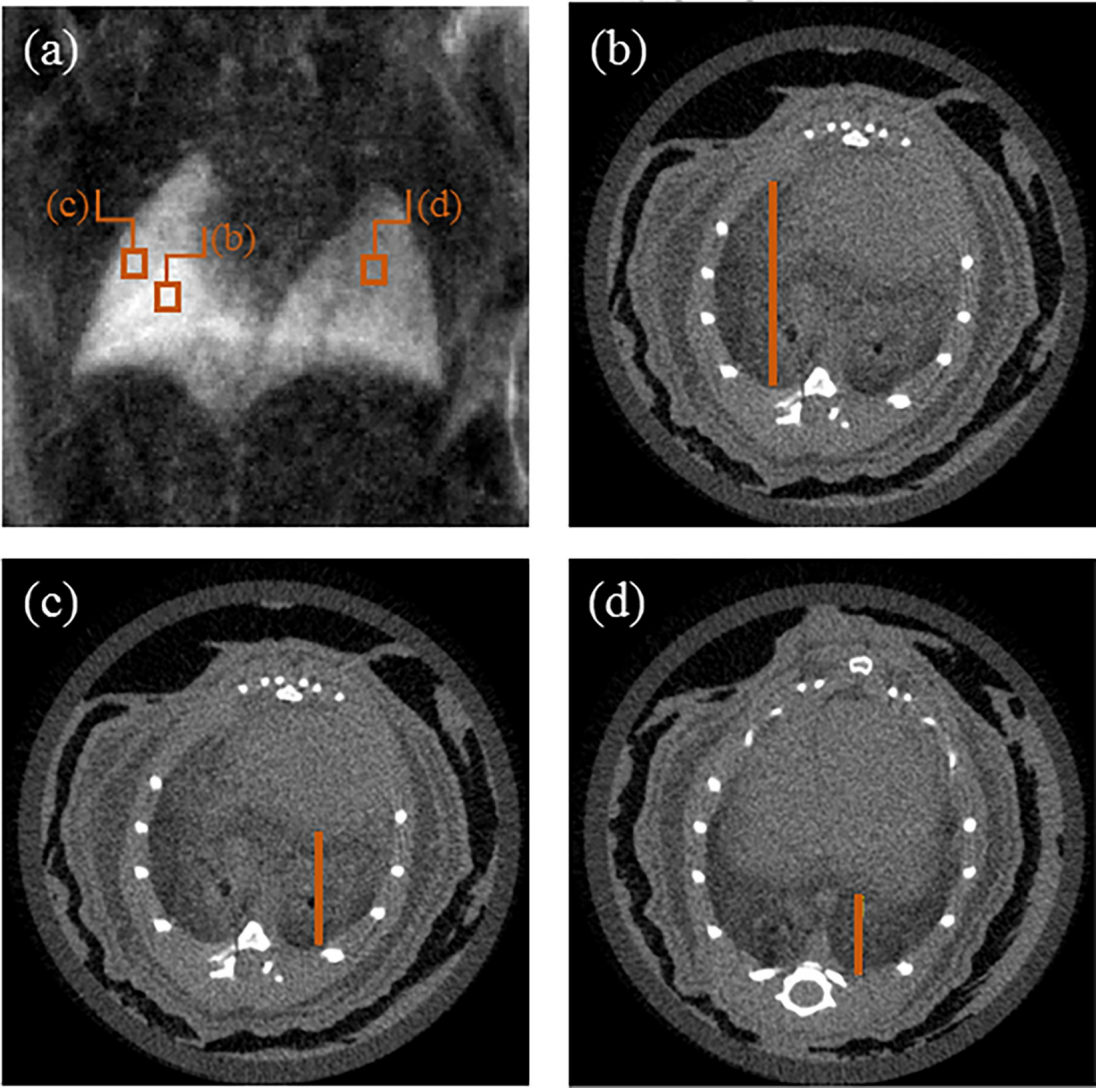
Dark field (GI) scan (a) and *μ*CT slices (b-d) of the same mouse. Three regions of interest were selected in the dark field image and related with lung thickness in the *μ*CT scan.

In the experimental DF scan of the mouse (Fig 2), respective signals of 0.34±0.03, 0.44±0.03 and 0.58±0.02 were measured in the three regions (Fig 2B–2D) corresponding to a lung thickness of 8.0±0.2 mm, 5.7±0.2 mm and 4.0±0.3 mm or estimated *ϵ* values of (1.44±0.11)⋅10^−11^ mm^-1^, (1.54±0.15)⋅10^−11^ mm^-1^ and (1.43±0.14)⋅10^−11^ mm^-1^. This can only be an approximation since e.g. the mouse fur (even with ultrasound gel) and other anatomical features also cause DF signal loss. However, these values lie within the predicted range (Fig 1(A)) and thus support the mouse model and the framework used.

## Discussion

This work estimated the feasibility of human lung imaging using dark field radiography based on a simulation study. The results showed a strong decrease in the linear diffusion coefficient when going from murine to human lung models. While this could be interpreted as a loss of sensitivity when trying to apply DF imaging to human lungs, this is in fact good news. If the linear diffusion coefficient of human lung tissue were as large as that of murine lungs, DF contrast would be saturated after just 2 cm of lung tissue and it would be impossible to differentiate between different regions or pathological stages of the lung. This study therefore suggests the applicability of DF imaging for human lung and that the current interest in this topic is well founded.

Both the simulation platform, using the simulated monochromatic plane wave, and the lung model (single diameter spherical alveoli for a given simulation run, excluding bone, soft tissue and skin) are obviously simplified representations of reality. Still, the results are consistent with experimental data, supporting the approximations made. The linear diffusion coefficient is determined up to a range of magnitudes, between a specified minimum and maximum alveoli size. More accurate modelling of the lung tissue could narrow down this range and enable studies on the effect of pathologies and inflation on the DF response. The exact *ϵ* values will probably vary somewhat between systems due to different magnification and focal spot blur [21], however, for high frequency fluctuations like the alveoli, the influence of these pseudo-dark field effects is expected to be inferior. Beam hardening, on the other hand, may affect the measured DF signal as the mean energy of the spectrum is shifted away from the design energy of the setup [43,44]. In human imaging, the ribs are more pronounced than in mice and the effects of beam hardening will be stronger.

The framework described here, including these results, could have potential use in the optimization of GI setups for human lung imaging. During optimizing, there is always a trade-off between sensitivity and system visibility. Since the response of DF for human lungs is expected to be relatively large, mainly due to the lung thickness, an increase in G_2_ pitch could be considered. This would decrease the sensitivity of the setup but facilitate a higher system visibility and therefore reduce noise in the DF image. This would be opportune since achieving a high system visibility at high energies is challenging. Here, a mean energy of the chest RX was assumed to be 64.5 keV. At such high energies the system needs to be retuned with adapted gratings whose production is technically demanding, resulting in a visibility loss. Possible systems could be based on high-energy setups presented in the literature [45]. Note that the effective mean energy for DF depends on the system visibility versus energy and can deviate from the mean energy assumed. Moreover, the high energy in chest RX was chosen based on current practice in transmission imaging, but it may be desirable to decrease the applied energy in order to increase the dark field contrast. Furthermore, following Lynch et al [18], the setup could be further tuned towards the larger diameter alveoli.

## Conclusion

This simulation study has demonstrated that it is possible to replicate the experimental dark field response of murine lungs. The model was applied on human lungs and predicted a linear diffusion coefficient some 100 times lower than that found for murine lungs. Since human lungs are much thicker than murine lungs, viable, practically useful dark field values can still be generated using a standard GI setup. Therefore, this study supports the translation of dark field murine lung imaging to chest applications in humans. In the future, similar simulation studies could help to optimize GI setups for human dark field lung imaging.
